# Psychometric evaluation of the Arabic brief forms of the diabetes distress scale in Egyptian patients with type II diabetes

**DOI:** 10.1186/s42506-026-00216-3

**Published:** 2026-04-29

**Authors:** Hazem A. Sayed Ahmed, Samaa I. Taha, Rehab A. Mohamed, Mahmoud A. Moussa, Amira E. Elfouly

**Affiliations:** 1 Department of Family Medicine, Faculty of Medicine, Suez Canal University, Ismailia, Egypt; 2 Family and Community Medicine Department, Ibn Sina National College for Medical Studies, Jeddah, Saudi Arabia; 3 Department of Psychology, Faculty of Education, Suez Canal University, Ismailia, Egypt

**Keywords:** Diabetes distress, DDS-2, DDS-4, Family medicine settings, Psychometric evaluation, Type 2 diabetes mellitus

## Abstract

**Background:**

Diabetes distress is highly prevalent among patients with type 2 diabetes mellitus (T2DM), and is consistently associated with poor self-care, medication nonadherence, and suboptimal glycemic control, leading to worse outcomes and reduced quality of life. Despite its clinical significance, routine screening is often overlooked in primary care and family medicine settings due to limited time and resources. Brief tools, such as the 2-item (DDS-2) and 4-item (DDS-4) Diabetes Distress Scales, offer practical alternatives to longer measures; however, validated Arabic versions are lacking. Given the high burden of T2DM in the Middle East and North Africa, culturally adapted and reliable screening instruments are necessary. This study evaluated the psychometric properties of the Arabic DDS-2 and DDS-4 among Egyptian patients with T2DM.

**Methods:**

A cross-sectional study was conducted with 366 participants recruited from five urban family medicine settings in Port Said, Egypt. Internal consistency was evaluated using Cronbach’s α, and confirmatory factor analysis (CFA) was used to assess the one-factor structure of the Arabic DDS-4. Concurrent, convergent, and predictive criterion validity were assessed by examining correlations between the Arabic DDS-2 and DDS-4 and the 17-item Diabetes Distress Scale (DDS-17), Patient Health Questionnaire-9 (PHQ-9), General Medication Adherence Scale (GMAS), and glycated hemoglobin (HbA1c). Known-group validity was assessed through associations with sociodemographic and clinical characteristics. Criterion validity was evaluated using receiver operating characteristic (ROC) curves, with high diabetes distress defined by the DDS-17 cutoff (≥ 3).

**Results:**

The Arabic DDS-2 and DDS-4 demonstrated strong internal consistency, with Cronbach’s alpha values of 0.728 and 0.904, respectively. CFA confirmed a one-factor structure for the Arabic DDS-4. The Arabic DDS-2 and DDS-4 showed very strong correlations with the DDS-17 (*r* = 0.910–0.953, *p* < 0.001), supporting strong concurrent validity. Both scales demonstrated moderate positive correlations with depressive symptoms (*r* ≈ 0.53–0.55, *p* < 0.001) and weak negative correlations with medication adherence (*r* ≈ − 0.31 to − 0.33, *p* < 0.001), indicating good convergent validity. Their weak correlations with HbA1c (*r* ≈ 0.19–0.21, *p* < 0.001) provided evidence of fair predictive validity. Known-group validity was confirmed by higher distress scores among patients with lower education, physical inactivity, failure to achieve glycemic targets, insulin use, complications, comorbidities, depressive symptoms, and poor medication adherence (all *p* < 0.05). The area under the curve (AUC) was higher for the DDS-4 (0.958) than for the DDS-2 (0.846), indicating stronger discriminative ability. The DDS-2 achieved excellent specificity (99.6%) and positive predictive value (98.6%), whereas the DDS-4 showed superior sensitivity (95.9%) and negative predictive value (98.9%).

**Conclusion:**

The Arabic DDS-2 and DDS-4 are reliable and valid instruments for screening diabetes distress in Arabic-speaking patients with T2DM. Their brevity makes them particularly useful in busy clinical settings, while their psychometric strength supports application in diverse healthcare contexts and research.

**Supplementary Information:**

The online version contains supplementary material available at 10.1186/s42506-026-00216-3.

## Introduction

Diabetes has been a global health issue in the past, is a significant concern now, and will continue to be in the future. In 2021, global diabetes affected 536.6 million people (10.5%), projected to rise to 783.2 million (12.2%) by 2045. The prevalence in the Middle East and North Africa (MENA) was higher at 16.2% (72.7 million), expected to reach 19.3% (135.7 million) by 2045. Egypt ranked 10th with 20.9% in 2021 and is projected to remain 10th with 23.4% in 2045. Over 90% of individuals with diabetes have type 2 diabetes mellitus (T2DM) [1].

Diabetes distress, introduced in 1995 by psychologists and psychiatrists from the Joslin Diabetes Center, encompasses the emotional stress and negative emotions associated with managing diabetes, addressing both psychological and practical challenges [2]. A meta-analysis indicates that 36% of individuals with T2DM experience diabetes distress. While its prevalence among primary care patients with T2DM ranges from 1.2% to 24.4% worldwide, it is 13.4% in Egypt [3]. This distress is associated with reduced medication adherence, poorer glycemic control, and diminished quality of life in T2DM patients [3–5].

Medication adherence is crucial for achieving optimal glycemic control in patients with T2DM. In Egypt, suboptimal adherence to antidiabetic medications remains a challenge in primary care, with reported rates ranging from 20.06% to 38.9% [6, 7]. Psychosocial factors, including diabetes distress, contribute to poor adherence, and addressing these factors can improve the quality of diabetes care [8].

National and international guidelines recommend regular monitoring for diabetes distress, particularly when treatment goals are unmet or complications arise, and advise referral to qualified mental health professionals when necessary [2, 8].

Polonsky and colleagues in the United States developed the 20-item Problem Areas in Diabetes (PAID) scale to assess diabetes distress. They later refined it into the 17-item Diabetes Distress Scale (DDS-17) [9, 10]. Both scales are widely used to evaluate diabetes distress in people with T2DM [11]. While the PAID focuses on emotional concerns related to food and complications, the DDS-17 emphasizes physician-related distress and self-management challenges. Both scales demonstrate a four-factor structure and confirmed reliability. The PAID is linked to dysfunctional coping, quality of life, and depression, whereas the DDS-17 is associated with improved self-care and metabolic outcomes [11].

The DDS-17 comprises four subscales: emotional burden, physician-related distress, regimen-related distress, and interpersonal distress [10]. The psychometric evaluation of the DDS-17 has been conducted in multiple countries, including the development of two Arabic versions for use among Jordanians and Saudis [12–15].

Fisher and colleagues developed two brief tools for screening diabetes distress: the 2-item Diabetes Distress Scale (DDS-2) and the 4-item Diabetes Distress Scale (DDS-4). Both tools include items from the emotional burden and regimen-related distress subscales of the DDS-17. Both DDS-2 and DDS-4 demonstrated high accuracy (96.7%) with a 3.3% false-negative rate. The DDS-4 showed only a minimal advantage in reducing false positives (15.1% vs. 13.7%, Δ = 1.4%). Fisher et al. argued that this marginal gain may not justify the added time, recommending the DDS-2 as the preferred initial screening tool, particularly in busy clinical settings. Patients screening positive (average ≥ 3 or sum ≥ 6) can then complete the full DDS-17, which identifies distress across its four domains. Reviewing items scored ≥ 3 helps facilitate targeted discussions, enabling clinicians to address specific patient concerns efficiently and develop tailored care plans [16].

The DDS-2 is more commonly used than the DDS-4 among patients with both type 1 and type 2 diabetes [13, 17–24]. However, only a limited number of studies have assessed the psychometric properties [13] and clinical utility of these brief measures across different cultures, ethnic groups, and clinical settings. Most studies have utilized the English version of the DDS-2 [19, 20, 23, 24] and DDS-4 [21]. Only two studies have employed translated versions of the DDS-2 (Malay, Chinese, and Norwegian); these lacked rigorous psychometric validation [18, 22].

Although diabetes distress is highly prevalent among Egyptian patients [3], it remains under-recognized in family medicine settings, where time constraints limit the use of longer instruments like the DDS-17 and PAID. While Arabic versions of the PAID and the 5-item PAID have been validated and shown to be reliable in Egyptian primary care [25, 26], no brief Arabic forms of the DDS-17 were previously available. Therefore, translating and validating the DDS-2 and DDS-4 into Arabic is essential to provide practical and reliable tools for early screening of diabetes distress. This study assessed the psychometric properties of these scales among Egyptian patients with T2DM in family medicine settings.

## Methods

### Study setting, design, and sampling

This cross-sectional study was conducted across five urban family medicine settings in Port Said, Egypt, from June 2022 to June 2023. Soper’s online calculator was used to determine the minimum required sample size of 200 participants for factorial validation of the DDS-2 and DDS-4, based on an effect size (f^2^ = 0.1), 90% power, and a 95% confidence level [27]. Based on our knowledge and the absence of prior reports on the exact proportion of this problem in comparable populations, we selected a small-to-medium effect [28] to provide a conservative estimate and to ensure sufficient power to detect even modest factor loadings in these brief scales. An additional 20% was added for potential non-response, bringing the required sample size to 240, with an actual sample size of 366.

A convenience sampling strategy was used to recruit eligible patients. Participants were Egyptian adults aged 18 or older who had been diagnosed with T2DM for at least one year and provided informed consent. Exclusion criteria included pregnant women with gestational diabetes, patients with severe medical conditions (such as end-stage renal disease, congestive heart failure, or decompensated liver failure), and individuals with significant cognitive or hearing impairments that could hinder their ability to understand or complete the questionnaire.

### Data collection tool

Data were collected using an anonymous structured questionnaire administered in face-to-face interviews by the co-first author. Each interview, lasting approximately 20 min on average, collected demographic information, lifestyle, and disease profile, and included the Arabic versions of the DDS-2, DDS-4, DDS-17, the Patient Health Questionnaire-9 (PHQ-9), and the General Medication Adherence Scale (GMAS) [10, 15, 16, 29–31]. No incentives were offered to participants for their participation.

The first section of the questionnaire collected sociodemographic data, including age, gender, education, employment, and income. The second section addressed lifestyle and clinical characteristics, including diabetes duration, comorbidities, complications, medications, and family history of diabetes. Lifestyle factors, smoking status, and regular physical activity, defined as at least 150 min of moderate- to vigorous-intensity aerobic exercise per week, spread over a minimum of three days with no more than two consecutive days without activity; for younger and physically fit individuals, at least 75 min per week of vigorous or interval training was considered sufficient [8].

The DDS-2 is a brief, two-item version of the DDS-17. Participants rated feelings of being “overwhelmed” and “failing” in diabetes management over the past month on a scale from 1 (no distress) to 6 (serious distress), with distress categorized as low (< 3) or high (≥ 3). The DDS-2 has shown good reliability (Cronbach’s alpha = 0.75–0.79) and strong psychometric properties [12, 16, 19].

The DDS-4 assessed diabetes distress over the past month. Participants rated four items from 1 (not a problem) to 6 (serious problem), including feeling overwhelmed by diabetes, often failing in their regimen, lacking motivation for self-management, and feeling angry, scared, or depressed about living with diabetes. The DDS-4 score is the average of these four items, with a mean score of ≥ 3 indicating high distress [16]. This scale has shown strong reliability (Cronbach’s alpha 0.88) [21].

The DDS-17 includes 17 questions across four areas: emotional burden (5 items), physician-related distress (4 items), regimen-related distress (5 items), and interpersonal distress (3 items). Responses are rated on a 6-point Likert scale, with distress levels categorized as low (< 3), moderate (3–3.9), and high (≥ 4). It has a Cronbach’s alpha of 0.93 [10].

The Arabic DDS-2 and DDS-4 were derived from the Arabic DDS-17, based on the Saudi DDS-17. The Saudi DDS-17 is a valid instrument with a Cronbach’s alpha of 0.848 [15]. We modified some wording for cultural adaptation, such as “my doctor doesn’t give me clear enough directions on how to manage my diabetes” (Item 4), “not testing my blood sugars” (Item 5), “failing with my diabetes regimen” (Item 6), “self-care efforts” (item 7), and “my diabetes self-management” (Item 16). The senior author initially translated the original English version into Arabic (forward translation), and another bilingual translator (the principal investigator) independently translated it back to English (backward translation). Both translations were then compared for consistency in meaning. This process was repeated by additional bilingual translators (co-first and third authors) until no discrepancies remained.

Our Arabic DDS-17 was initially psychometrically evaluated in a sample of Egyptian patients with T2DM, demonstrating excellent reliability (Cronbach’s α = 0.977). Reliability for each subscale was also high: emotional burden (α = 0.938), physician-related distress (α = 0.857), regimen-related distress (α = 0.928), and interpersonal-related distress (α = 0.951). Factor analysis supported the expected four-factor structure of our Arabic DDS-17. The Arabic DDS-17 score showed a significant positive correlation with depressive symptoms (Arabic PHQ-9) and a negative correlation with medication adherence (Arabic GMAS), indicating satisfactory convergent validity. The Arabic DDS-17 demonstrated discriminant validity by differentiating diabetes distress based on education, complication, comorbidities, glycemic control, depressive symptoms, and medication adherence (Supplementary file 1).

The PHQ-9 consists of 9 items scored from 0 (not at all) to 3 (nearly every day). A score of ≥ 10 indicates major depression with 88% sensitivity and specificity. The PHQ-9 is valid and reliable, with a Cronbach’s alpha of 0.89 [29], and the Arabic version has a Cronbach’s alpha of 0.857 [30].

The GMAS was originally developed in Urdu and later validated in English and Arabic. It includes 11 items with responses scored from 0 (always) to 3 (never). The total score ranges from 0 to 33, with a cutoff of 27 for adherence. The Arabic GMAS has a Cronbach’s alpha of 0.865 [31].

Anthropometric measurements, including body weight (kg) and height (cm), were obtained from all participants. Body mass index (BMI) was calculated as weight in kilograms divided by height in meters squared. Participants with a BMI of 18.5–24.9 kg/m² were classified as normal weight, those with a BMI of 25–29.9 kg/m² as overweight, and those with a BMI of 30 kg/m² or higher as obese. The most recent glycated hemoglobin (HbA1c) results (within the past 3 months) were recorded. Glycemic control is deemed good if HbA1c is less than 7% for adults or less than 7.5% for older adults [8].

### Statistical analysis

Data were analyzed using the Statistical Package for the Social Sciences (SPSS), version 25.0 (IBM Corporation, NY, USA), and Mplus version 7.4. Categorical variables were presented as frequencies and percentages, while continuous variables were assessed for normality using the Shapiro–Wilk test and reported as median and interquartile range when non-normally distributed. Reliability of the Arabic DDS-2 and DDS-4 was evaluated using Cronbach’s alpha. Confirmatory factor analysis (CFA) with robust weighted least squares estimation examined their one-factor structure. Model fit was assessed by the Chi-square [χ²] to degrees of freedom [df] ratio (CMIN/DF) (< 3), Tucker Lewis Index (TLI) and comparative fit index (CFI) (≥ 0.90), and root mean squared error of approximation (RMSEA) (≤ 0.08) [32].

Convergent validity was assessed through Pearson’s correlation (r) between the Arabic DDS-2 and DDS-4 with PHQ-9 and GMAS. Concurrent and predictive validity were evaluated by correlating DDS-2 and DDS-4 with DDS-17 and HbA1c, respectively. Correlation strengths were categorized as very weak (0–0.19), weak (0.20–0.39), moderate (0.40–0.59), strong (0.60–0.79), and very strong (0.80–1.0) [33]. Known-group validity was demonstrated by the ability to distinguish between groups based on depressive symptoms, adherence, and glycemic control. Criterion validity was assessed by calculating sensitivity, specificity, positive predictive value (PPV), and negative predictive value (NPV), using a DDS-17 score of ≥ 3 to indicate high distress [10, 16].

Criterion validity was assessed using receiver operating characteristic (ROC) curves, with high diabetes distress defined according to the DDS-17 cutoff of ≥ 3 [10, 16]. For both DDS-2 and DDS-4, the optimal cutoff point was determined, and the corresponding area under the curve (AUC), sensitivity, specificity, PPV, and NPV were reported.

## Results

### Characteristics of the study population (*N*=366)

The study included 366 patients with T2DM, with a mean age of 57.06 years (± 14.02). Among participants, 56.5% were female, and 79% were married. Over half (52.7%) reported insufficient income. Complications were reported in 16.9% of participants with one complication and in 29.5% with two or more. Additionally, 54.4% reported having two or more comorbid conditions. Most participants were classified as obese (69.7%), followed by overweight (29.0%), while only a small proportion had a normal BMI (1.4%) (Table 1).

**Table 1:** Sociodemographic and clinical characteristics of the study participants (*N* = 366)

Characteristics	Frequency (percent)
Age (years)	
Adults < 65 years	247 (67.5)
Older adults ≥ 65 years	119 (32.5)
Gender	
Male	169 (46.2)
Female	197 (53.8)
Marital status	
Unmarried	77 (21)
Married	289 (79)
Educational level	
Illiterate / can read and write	100 (27.3)
Primarily to secondary school educated	162 (44.3)
University graduate or above	104 (28.4)
Occupation	
Not working or a housewife	180 (49.2)
Manual worker	42 (11.5)
Trades, semiprofessional, and professional	144 (39.3)
Income (patients’ perceptions)	
Not Sufficient	193 (52.7)
Sufficient	173 (47.3)
Smoking	
Current smoker	65 (17.8)
Never smoker	269 (73.5)
Ex-smoker	32 (8.7)
Regular physical activity (Active)	119 (32.5)
Diabetes duration (years)	
≤ 5 years	148 (40.4)
6–10 years	119 (32.5)
> 10 years	99 (27)
Diabetes complications	
No complication	196 (53.6)
One complication	62 (16.9)
≥2 complications	108 (29.5)
Retinopathy (present)	73 (19.9)
Nephropathy (present)	85 (23.2)
Peripheral neuropathy (present)	39 (10.7)
Autonomic neuropathy (present)	28 (7.7)
Cardiovascular disease (present)	57 (15.6)
Comorbidities	
No comorbidity	1 (0.3)
One comorbidity	166 (45.4)
≥2 comorbidities	199 (54.4)
Hypertension (present)	167 (45.6)
Dyslipidemia (present)	119 (32.5)
Overweight or obesity (present)	361 (98.6)
Family history of diabetes (present)	229 (62.6)
Antidiabetic medications	
Oral hypoglycemic drugs	244 (66.7)
Insulin	52 (14.2)
Oral hypoglycemic drugs plus insulin	70 (19.1)
Glycemic control	
Uncontrolled	247 (67.5)
Controlled	119 (32.5)

### Reliability of the Arabic DDS-2 and DDS-4

The Arabic DDS-2 demonstrated a moderate correlation between item 1 and item 2 (*r* = 0.573, *p* < 0.001), with item-to-total correlations ranging from 0.882 to 0.891. The Cronbach’s alpha was 0.728. For the Arabic DDS-4, inter-item correlations ranged from 0.573 to 0.807, and item-to-total correlations varied from 0.815 to 0.933. The Cronbach’s alpha for the DDS-4 was 0.904 (Table 2).

**Table 2:** Internal consistency the Arabic DDS-2 and DDS-4 (*N* = 366)

Items of the Arabic DDS-2	Inter-items correlations (*N* = 366)			
	item 1		item 2	
Item 1				
Item 2	0.573*			
Arabic DDS-2 score	0.882*		0.891*	
	Cronbach α = 0.728			

Items of the Arabic DDS-4	Inter-items correlations (*N* = 366)			
	item 1	item 2	item 3	item 4
Item 1				
Item 2	0.573*			
Item 3	0.684*	0.788*		
Item 4	0.624*	0.728*	0.807*	
Arabic DDS-4 score	0.815*	0.879*	0.933*	0.894*
	Cronbach α = 0.904			

Item 1, “Feeling overwhelmed with the demands of living with diabetes”; Item 2, “Feeling that I am often failing with my diabetes routine”; Item 2, “Not feeling motivated to keep up my diabetes self-management”; Item 4, “Feeling angry, scared, and/or depressed when I think about living with diabetes”

DDS-2. 2-item Diabetes Distress Scale; DDS-4. 4-item Diabetes Distress Scale

Pearson correlation tests were conducted

*Statistically significant at p-value < 0.05

### Validity of the Arabic DDS-2 and DDS-4

#### Factor structure

**Fig. 1:**
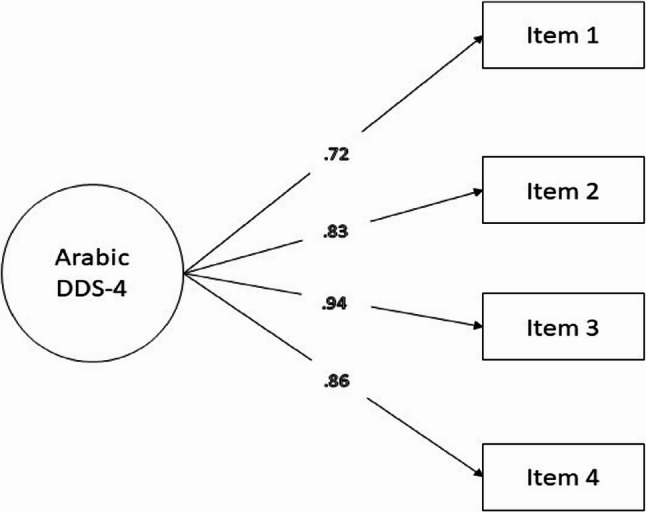
A path diagram illustrating the factor structure of the Arabic DDS-4 with item-specific factor loadings (*N* = 366)

A CFA was conducted for the Arabic DDS-4, as shown in Fig. 1 and detailed in Table 3. The factor loadings for the Arabic DDS-4 were strong, with significant values (*p* < 0.001) ranging from 0.717 to 0.941. Model fit indices indicate excellent fit: χ²(2) = 2.97, CMIN/DF = 1.48, CFI = 0.999, TLI = 0.997, SRMR = 0.008, and RMSEA = 0.036. The CFA results further indicated that no post-hoc model modifications were required, as the original four-item structure provided excellent fit without the need for item removal, cross-loadings, or correlated residuals.

**Table 3:** Factor loadings of the Arabic brief DDS from the confirmatory factor analysis (*N* = 366)

Items of the Arabic DDS-4	Factor loadings		
	Standardized estimate	SE	*p*-value
Item 1	0.717	0.046	< 0.001
Item 2	0.834	0.043	< 0.001
Item 3	0.941	0.040	< 0.001
Item 4	0.859	0.425	< 0.001
	Goodness-of-fit indices		
Model fit χ^2^ (df, p-value)	2.97 (2, < 0.001*)		
CMIN/DF	1.48		
CFI	0.999		
TLI	0.997		
SRMR	0.008		
RMSEA	0.036		

Item 1, “Feeling overwhelmed with the demands of living with diabetes”; Item 2, “Feeling that I am often failing with my diabetes routine”; Item 2, “Not feeling motivated to keep up my diabetes self-management”; Item 4, “Feeling angry, scared, and/or depressed when I think about living with diabetes”

DDS-4. 4-item Diabetes Distress Scale

CMIN/DF, ratio of Chi-square [χ²] value to the degrees of freedom [df] (good if CMIN/DF < 3); CFI, comparative fit index (good fit ≥ 0.90); TLI, Tucker Lewis Index (good if ≥ 0.90); SRMR, standardized root mean square residual (good fit ≤ 0.08); RMSEA, root mean square error of approximation (acceptable fit ≤ 0.08); SE, standard error

* Statistically significant at p-value < 0.05

#### Convergent, concurrent, and predictive criterion validity

Table 4 presents the correlations between the scores of Arabic DDS-2, DDS-4, DDS-17, PHQ-9, GMAS, and HbA1c levels. The DDS-2 and DDS-4 showed very strong positive correlations with the DDS-17 (DDS-2: *r* = 0.910, *p* < 0.001; DDS-4: *r* = 0.953, *p* < 0.001). Both scales also had moderate correlations with the PHQ-9 (DDS-2: *r* = 0.533, *p* < 0.001; DDS-4: *r* = 0.545, *p* < 0.001). Weak negative correlations were found with the GMAS (DDS-2: *r* = -0.325, *p* < 0.001; DDS-4: *r* = -0.307, *p* < 0.001), and very weak to weak correlations with HbA1c (DDS-2: *r* = 0.190, *p* < 0.001; DDS-4: *r* = 0.208, *p* < 0.001).

**Table 4:** Correlation between the Arabic DDS-2, DDS-4, DDS-17, PHQ-9, GMAS and HbA1c (*N* = 366)

Variables	DDS-17 score	Emotional burden score	Physician-related distress score	Regimen-related distress score	Interpersonal-related distress score	PHQ-9 total score	GMAS total score	HbA1c
Item 1	0.780^**^	0.853^**^	0.615^**^	0.719^**^	0.688^**^	0.485^**^	-0.332^**^	0.101
Item 2	0.832^**^	0.694^**^	0.700^**^	0.890^**^	0.766^**^	0.460^**^	-0.247^**^	0.234^**^
Item 3	0.896^**^	0.733^**^	0.774^**^	0.912^**^	0.880^**^	0.497^**^	-0.251^**^	0.193^**^
Item 4	0.848^**^	0.758^**^	0.721^**^	0.820^**^	0.830^**^	0.478^**^	-0.255^**^	0.204^**^
DDS-2 score	0.910^**^	0.871^**^	0.743^**^	0.909^**^	0.821^**^	0.533^**^	-0.325^**^	0.190^**^
DDS-4 score	0.953^**^	0.861^**^	0.799^**^	0.950^**^	0.899^**^	0.545^**^	-0.307^**^	0.208^**^

Item 1, “Feeling overwhelmed with the demands of living with diabetes”; Item 2, “Feeling that I am often failing with my diabetes routine”; Item 2, “Not feeling motivated to keep up my diabetes self-management”; Item 4, “Feeling angry, scared, and/or depressed when I think about living with diabetes”

DDS-2. 2-item Diabetes Distress Scale; DDS-4. 4-item Diabetes Distress Scale; DDS-17. Diabetes Distress Scale 17; Glycated hemoglobin, HbA1c; GMAS, General Medication Adherence Scale; PHQ-9, Patient Health Questionnaire 9

** Pearson Correlation is significant at *p* < 0.001 level (2-tailed)

The Arabic DDS-2 and DDS-4 showed strong positive correlations with the emotional burden subscale (DDS-2: *r* = 0.871, *p* < 0.001; DDS-4: *r* = 0.861, *p* < 0.001) and the physician-related distress subscale (DDS-2: *r* = 0.743, *p* < 0.001; DDS-4: *r* = 0.799, *p* < 0.001). They also exhibited very strong correlations with the regimen-related distress subscale (DDS-2: *r* = 0.909, *p* < 0.001; DDS-4: *r* = 0.950, *p* < 0.001) and strong correlations with the interpersonal-related distress subscale (DDS-2: *r* = 0.821, *p* < 0.001; DDS-4: *r* = 0.899, *p* < 0.001).

#### Known-group validity

The Arabic DDS-2 and DDS-4 effectively distinguished diabetes distress levels across demographic and clinical groups. Higher distress scores on both scales were associated with lower education (*P* < 0.001, *P* < 0.001), physical inactivity (*P* = 0.021, *P* = 0.001), and uncontrolled glycemic targets (*P* < 0.001, *P* < 0.001). Patients on insulin, those with diabetes complications, or multiple comorbidities also reported higher scores (all *P* < 0.001). Known-group validity was confirmed by significant associations with depressive symptoms (PHQ-9 ≥ 10, *P* < 0.001) and poor medication adherence (GMAS ≤ 26, *P* < 0.001) for both scales. The median and interquartile range showed the same pattern as the mean, with higher distress levels in these groups (Table 5).

**Table 5:** Association of the Arabic DDS-2 and DDS-4 with sociodemographic and clinical characteristics among study participants (*N* = 366)

Characteristics	DDS-2			DDS-4		
	Mean (± SD)	Median (IQR)	*p*-value	Mean (± SD)	Median (IQR)	*p*-value
Educational Level						
Illiterate / can read and write	2.66 (1.14)	2.50 (2.00-3.25)	< 0.001*	2.47 (1.15)	2.00 (1.63–3.25)	< 0.001*
Primarily to secondary school educated	2.50 (1.30)	2.00 (2.00–3.00)		2.28 (1.33)	1.75 (1.50–2.75)	
University graduate or above	2.02 (1.10)	2.00 (1.00-2.50)		1.83 (1.01)	1.50 (1.13–2.13)	
Regular physical activity						
Active	2.16 (1.01)	2.00 (1.50–2.50)	0.021*	1.91 (0.96)	1.50 (1.25–2.25)	0.001*
Inactive	2.53 (1.30)	2.00 (1.50-3.00)		2.35 (1.31)	1.75 (1.50-3.00)	
Diabetes complications						
No complication	2.17 (1.18)	2.00 (1.50–2.50)	< 0.001*	1.96 (1.17)	1.50 (1.25-2.00)	< 0.001*
One complication	2.42 (1.09)	2.00 (1.50-3.00)		2.21 (1.07)	1.75 (1.50–2.75)	
≥2 complications	2.85 (1.28)	2.50 (2.00-3.50)		2.65 (1.29)	2.25 (1.75–3.50)	
Comorbidities						
No comorbidity	2.50 (0.0.00)	2.50 (2.50–2.50)	< 0.001*	3.25 (0.00)	3.25 (3.25–3.25)	< 0.001*
One comorbidity	2.21 (1.30)	2.00 (1.50–2.50)		2.02 (1.25)	1.50 (1.25–2.25)	
≥2 comorbidities	2.58 (1.15)	2.00 (2.00–3.00)		2.36 (1.18)	2.00 (1.50-3.00)	
Antidiabetic medications						
Oral hypoglycemic drugs	2.28 (1.15)	2.00 (1.50–2.50)	< 0.001*	2.07 (1.15)	1.75 (1.50–2.25)	< 0.001*
Insulin	2.44 (1.40)	2.00 (1.25–3.50)		2.31 (1.41)	1.63 (1.25-3.00)	
Oral hypoglycemic drugs plus Insulin	2.83 (1.29)	2.50 (2.00-3.50)		2.63 (1.25)	2.25 (1.75–3.50)	
Glycemic control						
Uncontrolled	2.61 (1.34)	2.00 (1.50–3.50)	< 0.001*	2.43 (1.34)	2.00 (1.50–3.25)	< 0.001*
Controlled	2.01 (0.83)	2.00 (1.50-2.00)		1.75 (0.75)	1.50 (1.50–1.75)	
Depressive symptoms (PHQ-9 ≥ 10)						
Absent	2.03 (0.98)	2.00 (1.50–2.50)	< 0.001*	1.82 (0.95)	1.50 (1.25-2.00)	< 0.001*
Present	3.38 (1.26)	3.50 2.50–4.50)		3.19 (1.30)	3.00 (2.00-4.25)	
Medication Adherence (GMAS ≥ 27)						
Suboptimal	2.53 (1.19)	2.00 2.00–3.00)	< 0.001*	2.30 (1.20)	1.75 (1.50–2.75)	< 0.001*
Optimal	2.01 (1.27)	1.50 1.00-2.50)		1.91 (1.26)	1.50 (1.00–2.00)	

DDS-2, 2-item Diabetes Distress Scale; DDS-4. 4-item Diabetes Distress Scale; DDS-17. Diabetes Distress Scale 17; IQR, Interquartile range; GMAS, General Medication Adherence Scale; PHQ-9, Patient Health Questionnaire 9

The Mann-Whitney and Kruskal-Wallis tests were performed

* *p* is significant at the level < 0.05

Age was not associated with significant differences in DDS-2 (*p* = 0.356) or DDS-4 scores (*p* = 0.120). Females reported higher distress scores than males, although the differences were not statistically significant (*p* = 0.693 and *p* = 0.795, respectively). Similarly, diabetes duration (*p* = 0.345 and *p* = 0.482) and BMI classifications (*p* = 0.266 and *p* = 0.226) showed no significant associations with either scale.

#### Criterion validity

**Fig. 2:**
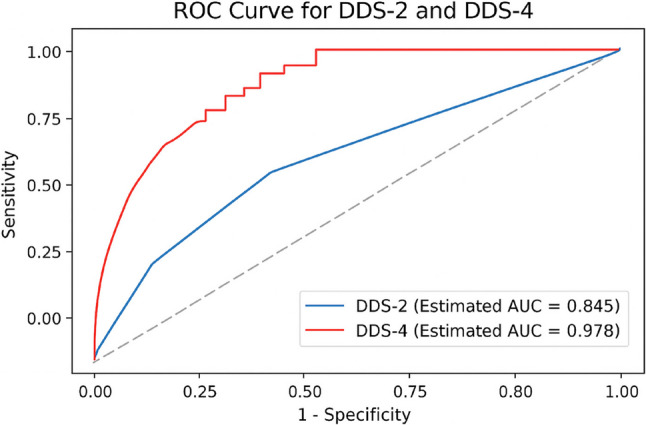
Receiver operating characteristic (ROC) curve for DDS-2 and DDS-4 (*N* = 366). AUC, Area Under the Curve; DDS-2, 2-item Diabetes Distress Scale; DDS-4. 4-item Diabetes Distress Scale; ROC, Receiver Operating Characteristic

Table 6; Fig. 2 show that both brief forms demonstrated strong diagnostic performance, with the DDS-4 achieving a higher AUC (0.958, 95% CI: 0.937–0.979) than the DDS-2 (0.846, 95% CI: 0.806–0.886). The DDS-2 demonstrated excellent specificity (99.6%) and PPV (98.6%), whereas the DDS-4 provided superior sensitivity (95.95%) and NPV (98.94%).

**Table 6:** Diagnostic performance of DDS-2 and DDS-4 compared with DDS-17 (*N* = 366)

Scale	Sensitivity (%)	Specificity (%)	Positive predictive value (%)	Negative predictive value (%)	Estimated area under the curve
DDS-2 (Cutoff ≥ 3)	69.5	99.6	98.6	89.0	0.845
DDS-4 (Cutoff ≥ 3)	95.95	95.55	84.52	98.94	0.978

DDS-2, 2-item Diabetes Distress Scale; DDS-4. 4-item Diabetes Distress Scale

Diabetes Distress Scale 17 (Cutoff ≥ 3) was used as the reference standard

## Discussion

This study is the first to assess the psychometric properties of the Arabic DDS-2 and DDS-4 in family medicine settings within the MENA region. These scales demonstrate satisfactory psychometric properties in patients with T2DM, including good reliability, satisfactory construct validity, confirmed convergent validity, clear known-group validity, and strong criterion validity.

The Arabic DDS-2 demonstrated good internal consistency, while the Arabic DDS-4 exhibited excellent internal consistency. Our findings align with previous studies, reporting Cronbach’s alpha values of 0.75 to 0.79 for the DDS-2 and 0.88 for the DDS-4 [11, 18, 19]. Additionally, strong construct validity was found for both scales. CFA confirmed the one-factor structure of the Arabic DDS-4 with excellent goodness-of-fit indicators. Although the CFA for the Arabic DDS-2 did not yield conclusive results, the moderate correlation between its two items, along with satisfactory internal consistency, suggests they are related to the same underlying construct.

In our study, strong and significant positive correlations were observed between the Arabic DDS-2, DDS-4, and DDS-17 scores, consistent with the findings of Fisher et al. [16]. Although the correlations with the DDS-17 were very high, this does not indicate conceptual redundancy. Instead, these findings support strong concurrent validity, with the brief forms offering efficient screening tools in busy primary care while the DDS-17 remains valuable for comprehensive assessment across multiple distress domains.

The Arabic DDS-2 and DDS-4 were derived from the emotional burden and regimen-related distress domains of the DDS-17. As expected, they demonstrated strong correlations with these domains in our study. They also showed significant correlations with physician-related and interpersonal distress. This may be because the core items of the brief forms capture feelings of being overwhelmed, frustrated, or unsuccessful in managing diabetes—experiences that extend beyond self-care and influence interactions with healthcare providers and family members. These findings suggest that, although brief, the DDS-2 and DDS-4 reflect broader aspects of diabetes distress, supporting their construct validity and reinforcing their clinical value as efficient tools for capturing multidimensional distress.

In this study, Arabic DDS-2 and DDS-4 scores showed positive correlations with depressive symptoms (Arabic PHQ-9) and negative correlations with medication adherence (Arabic GMAS), supporting their convergent validity.

The Arabic DDS-2 and DDS-4 were positively correlated with higher depressive symptoms, consistent with findings from studies that used the DDS-17 [34, 35]. In particular, DDS-2 items capture feelings of being overwhelmed and failing in diabetes management, which are closely aligned with depressive affect. Likewise, DDS-4 items encompass low motivation and negative emotions such as anger and fear, all of which are strongly linked to depression. These associations suggest that brief DDS forms effectively capture the psychological burden of diabetes, highlighting the bidirectional relationship in which depressive symptoms both result from and contribute to diabetes distress.

In addition, the Arabic DDS-2 and DDS-4 were significantly associated with lower medication adherence. This finding aligns with evidence from a recent randomized clinical trial indicating that emotional burden is an important barrier to treatment adherence [36]. Patients who feel overwhelmed, demotivated, or perceive themselves as failing are more likely to struggle with consistent treatment adherence, while regimen-related distress further obstructs adherence when patients feel they are not meeting expectations.

Together, these findings highlight that the brief DDS forms not only capture the emotional dimensions of distress but also reveal their behavioral consequences, reinforcing their clinical value in both psychological and adherence-related aspects of diabetes care.

Our findings showed a weak positive correlation between the Arabic DDS-2 and DDS-4 scores and HbA1c, indicating fair predictive validity for the brief scales. Previous studies have also reported associations of the DDS-2 with HbA1c in both T2DM [13, 16–19] and type 1 diabetes mellitus (T1DM) [23, 24], as well as between the original DDS-4 and HbA1c [16]. This modest association may be explained by the fact that HbA1c reflects average glycemic control over two to three months, whereas the DDS-2 and DDS-4 capture psychological distress over the past month. The difference in timeframes likely weakens the correlation. Moreover, HbA1c is influenced by multiple factors beyond psychological distress, including medication adherence, dietary patterns, physical activity, and individual differences in glucose metabolism. Therefore, although greater distress may contribute to poorer glycemic control, the relationship is multifactorial and not solely driven by distress alone.

Our study showed that the Arabic DDS-2 and DDS-4 effectively differentiate levels of diabetes distress based on demographic and clinical characteristics. Patients with poor glycemic control exhibited higher distress scores than those with good control. Additionally, known-groups validity revealed significant differences in DDS-2 and DDS-4 scores between patients with higher and lower depressive symptoms, as well as between those with optimal and suboptimal medication adherence.

Although subgroup analyses by gender and age were not conducted, our data showed no statistically significant association between age or gender and diabetes distress, and further subgrouping could reduce statistical power. Future studies with larger samples are needed to explore whether gender or age moderates the performance of the Arabic DDS-2 and DDS-4.

The study indicated that low education was associated with higher diabetes distress, possibly due to a lack of knowledge about diabetes management, increased complication risks, and ineffective coping strategies [15, 37, 38]. Additionally, physical inactivity was associated with higher DDS-2 and DDS-4 scores, aligning with a previous Saudi study using the DDS-17 [39]. Diabetes distress is more prevalent among physically inactive patients, likely reflecting poor glycemic control, weight gain, and an increased risk of mental health problems.

Diabetes distress often arises from diabetes complications [40]. In our study, higher Arabic DDS-2 and DDS-4 scores were associated with more complications, consistent with previous research [41]. Additionally, a longitudinal study indicated that a greater number of complications is directly linked to the persistence of diabetes distress over time [42]. Patients facing more diabetes-related complications experience increased disease burden, complex treatment regimens, fear of worsening health, diminished quality of life, and financial stress. Similarly, our findings revealed that higher Arabic DDS-2 and DDS-4 scores correlated with an increased number of comorbidities. Previous studies have also shown that more comorbidities are associated with the ongoing experience of diabetes distress [42, 43], as they complicate care, increase physical and emotional strain, and elevate anxiety regarding health deterioration and treatment burdens.

Our findings showed that patients treated with insulin, either alone or in combination with oral hypoglycemic agents, reported significantly higher Arabic DDS-2 and DDS-4 scores compared with those on oral agents alone. This pattern suggests that insulin therapy is associated with greater diabetes distress, with the highest distress observed in those receiving both insulin and oral medications. These results are consistent with previous studies linking insulin use to higher distress [44, 45]. Possible explanations include the increased complexity of insulin management, greater treatment burden, more advanced disease and complications, financial costs, and fear of hypoglycemia. Such factors may amplify feelings of frustration, guilt, and being overwhelmed, which are captured by the brief DDS scales.

Our study demonstrated distinct strengths for the Arabic DDS-2 and DDS-4. The DDS-2 showed excellent specificity and PPV, making it well-suited for confirming diabetes distress in clinical practice, especially when accurate case identification is prioritized. Conversely, the DDS-4 achieved higher sensitivity and NPV, highlighting its utility as a sensitive screening tool to minimize missed cases.

From a clinical perspective, this suggests complementary utility: the DDS-4 can be prioritized for broad screening to minimize overlooked cases, while the DDS-2 may serve as a confirmatory tool in settings where diagnostic precision is essential. Although the DDS-4 requires slightly more time to administer than the DDS-2, the added value lies in its ability to reduce false negatives and improve case detection. In primary care, where under-recognition of diabetes distress is common, this sensitivity advantage may justify the modest increase in time and resource use. Economically, the minimal extra burden of administering two additional items is unlikely to outweigh the clinical benefit of identifying more distressed patients earlier, which may prevent downstream complications and associated healthcare costs.

In light of these findings, we propose two practical pathways for integrating the Arabic brief DDS forms into routine care. The DDS-4, with its higher sensitivity and brevity, can be used as an initial screening tool, with positive cases followed by the full DDS-17 for a comprehensive assessment across all distress domains. Alternatively, the DDS-2, due to its brevity and high specificity, may serve as a rapid first-line screener in very busy primary care settings, with positive cases subsequently assessed using the DDS-17. This stepwise approach enables clinicians to balance sensitivity, specificity, and efficiency according to the clinical context.

### Limitations of the study

Our study has several limitations. The lack of randomization may limit generalizability. The study focused exclusively on patients with T2DM, so findings may not extend to those with T1DM. While internal consistency was assessed, test-retest reliability was not evaluated, leaving the temporal stability of the Arabic DDS-2 and DDS-4 unexamined. We did not conduct cross-validation or subsample analyses; larger studies are needed to investigate the potential effects of age and gender. The design did not allow assessment of changes after interventions, highlighting the need for longitudinal studies. Although self-report questionnaires were avoided due to limited literacy, face-to-face interviews may still be subject to social desirability bias. Finally, recall bias may have influenced data collection.

## Conclusion

The Arabic DDS-2 and DDS-4 instruments are reliable and valid for assessing diabetes distress in Egyptian patients with T2DM. They are effective for initial screening and have potential applicability in other Arabic-speaking populations. Additionally, these scales are suitable for diabetes distress research in Egypt and other Arabic-speaking countries. This study also lays the groundwork for future research on the use of the Arabic DDS-2 in screening T1DM patients and in broader clinical settings.

## Supplementary Information

Supplementary Material 1.

## Data Availability

The datasets used and/or analyzed during the current study are available from the corresponding author upon reasonable request.

## Abbreviations

ATT39: 39-item Attitudes to Diabetes Scale
AUC: Area under the curve
BMI: Body Mass Index
CFA: Confirmatory factor analysis
CFI: Comparative fit index
CI: Confidence interval
CMIN/DF: Ratio of Chi-square value to the degrees of freedom
DDS-17: 17-item Diabetes Distress Scale
DDS-2: 2-item Diabetes Distress Scale
DDS-4: 4-item Diabetes Distress Scale
df: Degrees of freedom
GMAS: General Medication Adherence Scale
HbA1c: Glycated hemoglobin
MENA: Middle East and North Africa region
NPP: Negative predictive value
PAID: Problem Areas in Diabetes
PHQ-9: Patient Health Questionnaire 9
PPV: Positive predictive value
RMSEA: Root mean squared error of approximation
ROC: Receiver operating characteristic
SPSS: Statistical Package for the Social Sciences
SRMR: Standardized root mean square residual
T1DM: Type 1 diabetes mellitus
T2DM: Type 2 diabetes mellitus
TLI: Tucker Lewis Index
χ^2^: Chi-square
