# Supplementary material for: Psychometric evaluation of the Arabic brief forms of the diabetes distress scale in Egyptian patients with type II diabetes
Source: J Egypt Public Health Assoc. 2026 Apr 29;101:14. doi: 10.1186/s42506-026-00216-3 (PMC13129166; doi:10.1186/s42506-026-00216-3)
Supplement: Supplementary file 1 — Supplementary Material 1. [file 42506_2026_216_MOESM1_ESM.docx]

**Table S1. Factor loadings of the 4-factor model of the Arabic-DDS-17 among Egyptians with T2DM (N = 224)**

| **Factor structure of the Arabic DDS-17** | |  | **Factor loadings** | | |
| --- | --- | --- | --- | --- | --- |
| **Factors** | **Items number** |  | Std. estimates | SE | *p*-value |
| **Emotional burden** | **Item 2** |  | 0.955 | 0.0102 | <0.001 |
|  | **Item 4** |  | 0.949 | 0.0104 | <0.001 |
|  | **Item 7** |  | 0.947 | 0.0131 | <0.001 |
|  | **Item 10** |  | 0.880 | 0.0179 | <0.001 |
|  | **Item 14** |  | 0.811 | 0.0234 | <0.001 |
| **Physician-related distress** | **Item 1** |  | 0.761 | 0.0267 | <0.001 |
|  | **Item 5** |  | 0.821 | 0.0222 | <0.001 |
|  | **Item 11** |  | 0.684 | 0.0352 | <0.001 |
|  | **Item 15** |  | 0.952 | 0.0144 | <0.001 |
| **Regimen-related distress** | **Item 3** |  | 0.912 | 0.0134 | <0.001 |
|  | **Item 6** |  | 0.887 | 0.0155 | <0.001 |
|  | **Item 8** |  | 0.695 | 0.0311 | <0.001 |
|  | **Item 12** |  | 0.933 | 0.0123 | <0.001 |
|  | **Item 16** |  | 0.965 | 0.0073 | <0.001 |
| **Interpersonal-related distress** | **Item 9** |  | 0.954 | 0.0087 | <0.001 |
|  | **Item 13** |  | 0.954 | 0.0100 | <0.001 |
|  | **Item 17** |  | 0.982 | 0.0085 | <0.001 |
|  |  |  | **Goodness-of-fit indices** | | |
|  | **Model fit,** χ^2^ (df), *p*-value |  | **299 (111).< 0.001** | | |
|  | **CMIN/DF** (χ²/df) |  | **2.694** | | |
|  | **CFI** |  | **0.999** | | |
|  | **TLI** |  | **0.998** | | |
|  | **SRMR** |  | **0.051** | | |
|  | **RMSEA** |  | **0.087** | | |

DDS-17, Diabetes Distress Scale 17; CMIN/DF, ratio of chi-square [χ²] value to the degrees of freedom [df] (good if CMIN/DF<3); CFI, comparative fit index (good fit ≥0.90); TLI, Tucker Lewis Index (good if ≥0.90); SRMR, standardized root mean square residual (good fit ≤0.08); RMSEA, root mean square error of approximation (acceptable fit ≤0.08); SE, standard error

*. Statistically significant at p-value <0.001

**Table S2. Correlation between the Arabic DDS-17, PHQ-9, GMAS and HbA1c (N = 224)**

| **Variables** | **PHQ-9 total score** | **GMAS total score** | **HbA1c** |
| --- | --- | --- | --- |
| **DDS-17 total score** | 0.524^**^ | -0.275^**^ | 0.128 |
| **Emotional burden total score** | 0.562^**^ | -0.318^**^ | 0.049 |
| **Physician-related distress total score** | 0.403^**^ | -0.219^**^ | 0.111 |
| **Regimen-related distress total score** | 0.516^**^ | -0.257^**^ | 0.169^*^ |
| **Interpersonal-related distress total score** | 0.463^**^ | -0.219^**^ | 0.145^*^ |

DDS-17. Diabetes Distress Scale 17; Glycated hemoglobin, HbA1c; GMAS, General Medication Adherence Scale; PHQ-9, Patient Health Questionnaire 9

**. Pearson Correlation is significant at p<0.01 level (2-tailed).

*. Pearson Correlation is significant at p<0.05 level (2-tailed).

| **Characteristics** | **Mean (±SD)** | **Median (IQR)** | **p-value** |
| --- | --- | --- | --- |
| **Educational Level** |  |  |  |
| Illiterate and Read and write | 2.73 (1.43) | 2.15 (1.79-3.21) | <0.001* |
| Primary to secondary school educated | 2.51 (1.42) | 1.76 (1.59-3.29) |  |
| University graduate or above | 1.94 (1.06) | 1.65 (1.35-2.00) |  |
| **Regular physical activity** |  |  |  |
| Active | 1.94 (0.92) | 1.65 (1.47-1.88) | <0.001* |
| Inactive | 2.63 (1.47) | 1.94 (1.65-3.41) |  |
| **Diabetes complications** |  |  |  |
| No complication | 2.13 (1.28) | 1.65 (1.47-1.94) | <0.001* |
| One complication | 2.43 (1.12) | 1.94 (1.65-2.94) |  |
| ≥2 complications | 2.89 (1.47) | 2.50 (1.88-3.59) |  |
| **Comorbidities** |  |  |  |
| No comorbidity | 2.82 (0) | 2.82 (2.82-2.82) | 0.016* |
| One comorbidity | 2.30 (1.52) | 1.71 (1.47-2.41) |  |
| ≥2 comorbidities | 2.50 (1.22) | 1.94 (1.65-3.15) |  |
| **Antidiabetic medications** |  |  |  |
| Oral hypoglycemic drugs | 2.30 (1.37) | 1.76 (1.59-2.53) | 0.109 |
| Insulin | 2.50 (1.36) | 1.88 (1.53-3.47) |  |
| Oral hypoglycemic drugs plus Insulin | 2.65 (1.32) | 2.15 (1.65-3.59) |  |
| **Glycemic control** |  |  |  |
| Uncontrolled | 2.62 (1.47) | 1.94 (1.65-3.41) | <0.001* |
| Controlled | 1.84 (0.75) | 1.65 (1.50-1.82) |  |
| **Depressive symptoms (PHQ-9≥10)** |  |  |  |
| Absent | 1.98 (1.06) | 1.65 (1.47-1.94) | <0.001* |
| Present | 3.43 (1.45) | 3.12 (2.18-4.71) |  |
| **Medication Adherence (GMAS≥27)** |  |  |  |
| Suboptimal | 2.47 (1.31) | 1.88 (1.65-3.00) | 0.003* |
| Optimal | 2.16 (1.55) | 1.59 (1.00-2.12) |  |

**Table S3. Association of the Arabic DDS-17 with sociodemographic and clinical characteristics among Study participants (N = 224)**

DDS-17. Diabetes Distress Scale 17; IQR, Interquartile range; GMAS, General Medication Adherence Scale; PHQ-9, Patient Health Questionnaire 9.

The Mann-Whitney and Kruskal-Wallis tests were performed.

* p is significant at the level <0.05
